# Variability of 128 schizophrenia-associated gene variants across distinct ethnic populations

**DOI:** 10.1038/tp.2016.260

**Published:** 2017-01-03

**Authors:** K Ohi, T Shimada, T Yasuyama, T Uehara, Y Kawasaki

**Affiliations:** 1 Department of Neuropsychiatry, Kanazawa Medical University, Ishikawa, Japan

## Abstract

Schizophrenia is a common polygenetic disease affecting 0.5–1% of individuals across distinct ethnic populations. PGC-II, the largest genome-wide association study investigating genetic risk factors for schizophrenia, previously identified 128 independent schizophrenia-associated genetic variants (GVs). The current study examined the genetic variability of GVs across ethnic populations. To assess the genetic variability across populations, the 'variability indices' (VIs) of the 128 schizophrenia-associated GVs were calculated. We used 2504 genomes from the 1000 Genomes Project taken from 26 worldwide healthy samples comprising five major ethnicities: East Asian (EAS: *n*=504), European (EUR: *n*=503), African (AFR: *n*=661), American (AMR: *n*=347) and South Asian (SAS: *n*=489). The GV with the lowest variability was rs36068923 (VI=1.07). The minor allele frequencies (MAFs) were 0.189, 0.192, 0.256, 0.183 and 0.194 for EAS, EUR, AFR, AMR and SAS, respectively. The GV with the highest variability was rs7432375 (VI=9.46). The MAFs were 0.791, 0.435, 0.041, 0.594 and 0.508 for EAS, EUR, AFR, AMR and SAS, respectively. When we focused on the EAS and EUR population, the allele frequencies of 86 GVs significantly differed between the EAS and EUR (*P*<3.91 × 10^−4^). The GV with the highest variability was rs4330281 (*P*=1.55 × 10^−138^). The MAFs were 0.023 and 0.519 for the EAS and EUR, respectively. The GV with the lowest variability was rs2332700 (*P*=9.80 × 10^−^^1^). The MAFs were similar between these populations (that is, 0.246 and 0.247 for the EAS and EUR, respectively). Interestingly, the mean allele frequencies of the GVs did not significantly differ between these populations (*P*>0.05). Although genetic heterogeneities were observed in the schizophrenia-associated GVs across ethnic groups, the combination of these GVs might increase the risk of schizophrenia.

## Introduction

Schizophrenia is a common, complex psychiatric disease with a lifetime prevalence of ~0.5–1%^1, 2^ and an estimated heritability of ~80%.^3^ The incidence of schizophrenia is uniform worldwide.^1, 4, 5^ Hundreds of common genetic variants (GVs) have been weakly implicated in the pathogenesis of schizophrenia.^6, 7^ Genome-wide association studies (GWASs), which examine millions of GVs, are powerful tools for identifying common susceptibility variants associated with complex disorders (including schizophrenia) across diverse populations. The largest GWAS in the Schizophrenia Working Group of the Psychiatric Genomics Consortium (GWAS PGC-II), including 36 989 patients with schizophrenia and 113 075 controls, has identified 128 linkage disequilibrium (LD)-independent variants across 108 genomic loci.^7^ However, most of these participants were of European (EUR) ancestry. The second most common ethnic population included case–control samples from East Asia (1866 cases and 3418 controls).

These 128 LD-independent schizophrenia-associated GVs contribute to the risk of schizophrenia across distinct populations. For example, the schizophrenia-associated GVs in the *ZNF804A*, *NRGN*, *VRK2* and *ITIH3/4* genes^7^ are found in both EUR^7, 8, 9^ and Asian^10, 11, 12^ patients with schizophrenia; however, the significance levels of these associations in Asian populations are marginal but not significant across the genome. In contrast, GV rs115329265 in the major histocompatibility complex region on chromosome 6, the most significantly associated GV in schizophrenia,^7^ is not polymorphic in the Japanese population, according to the 1000 Genomes Project (1000GP: http://browser.1000genomes.org/index.html). This finding appears to contradict the evidence, suggesting that schizophrenia affects ~0.5–1% of individuals across distinct populations. We hypothesized that the sum of the allele frequencies of the 128 GVs would not differ across ethnic populations; however, the frequencies of each GV would differ across populations.

The human genome consists of three billion bases and over 88 million GVs (including 84.7 million single-nucleotide polymorphisms (SNPs), 3.6 million short insertions/deletions (indels) and 60 000 structural variants), which can differ between any two genomes in different people.^13^ The 1000GP, which was conducted between 2008 and 2015, sought to study these variations in many people; in doing so, it has provided a solid foundation upon which to build understanding of the genetic variation in humans.^13, 14, 15^ The 1000GP consortium has analyzed 2504 genomes across 26 populations from five continental regions (East Asians (EAS), EUR, Africans (AFR), Americans (AMR) and South Asians (SAS)), by using a combination of low-coverage whole-genome sequencing, deep exome sequencing and dense microarray genotyping. The 1000GP has demonstrated that a typical genome differs from the reference human genome at between 4.1 million and 5.0 million sites.^15^ The total number of observed non-reference sites differs greatly across populations.^15^ Individuals of AFR ancestry harbor the greatest number of variant sites among the five ethnic populations. In addition, individuals from recently admixed populations show great variability in the number of variants. The present study tested the genetic variability of the 128 LD-independent schizophrenia-associated GVs, including SNPs and indels, detected by using the most recently available data from the GWAS PGC-II^7^ with regard to the five ethnic populations, particularly EAS and EUR, studied in the 1000GP.

## Materials and methods

### Participants

The 1000GP is the largest public catalog of human variation and genotype data, comprising 2504 human genomes from 26 ethnic populations.^13, 14, 15^ The healthy individual genomes are divided into five major ethnic populations, EAS (*n*=504), EUR (*n*=503), AFR (*n*=661), AMR (*n*=347) and SAS (*n*=489), which were included in the current study and accessed via the 1000GP Phase 3 Browser (http://browser.1000genomes.org/index.html). EAS consists of Chinese Dai in Xishuangbanna, China (CDX); Han Chinese in Bejing, China (CHB); Southern Han Chinese (CHS); Japanese in Tokyo, Japan (JPT); and Kinh in Ho Chi Minh City, Vietnam (KHV). EUR consists of Utah residents with northern and western European Ancestry (CEU), Finnish in Finland (FIN), British in England and Scotland (GBR), Iberians in Spain (IBS); and Toscani in Italy (TSI). AFR consists of African Caribbeans in Barbados (ACB); Americans of African Ancestry in Southwest USA (ASW); Esan in Nigeria (ESN); Luhya in Webuye, Kenya (LWK); Mandinka in the Gambia (MAG); Mende in Sierra Leone (MSL); and Yoruba in Ibadan, Nigeria (YRI). AMR consists of Colombians from Medellin, Colombia (CLM); Mexicans from Los Angeles USA (MXL); Peruvians from Lima, Peru (PEL); and Puerto Ricans from Puerto Rico (PUR). SAS consists of Bengali from Bangladesh (BEB); Gujarati Indians from Houston, TX (GIH); Indian Telugu from the UK (ITU); Punjabis from Lahore, Pakistan (PJL); and Sri Lankan Tamils from the UK (STU). Demographic information for the participants is shown in Supplementary Table 1. According to the previous largest GWAS,^7^ the 128 LD-independent schizophrenia-associated GVs were extracted from these populations using the 1000GP Phase 3 Browser.

### Statistical analyses

All statistical analyses were performed using SPSS 21.0 (IBM SPSS Japan, Tokyo, Japan) and R 3.1.1 (http://www.r-project.org/). We defined a variability index (VI) to investigate the genetic variability of the 128 LD-independent schizophrenia-associated GVs among the five ethnic populations by using the following formula:


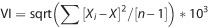


where X_*i*_ represents each minor allele frequency (MAF) weighted for the sample size (number of minor alleles (sqrt)) in each ethnic population and 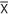 represents each mean MAF weighted for the sample size among the five ethnic populations. A high VI indicates high genetic variability among the ethnic populations, whereas a low VI indicates low genetic variability among the populations. The mean VI among the chromosomes was analyzed using analysis of variance with the VI as the dependent variable and chromosomes as the independent variable. To compare the genetic variability of the schizophrenia-associated GVs between the EAS and EUR populations that were identical to individuals utilized to calculate the VI, the differences were analyzed using *χ*^*2*^ or Fisher’s exact tests. The mean allele frequencies of the GVs between these populations were analyzed using non-parametric Mann–Whitney *U*-tests. To control for type I error (that is, false-positives), *P*-values less than 3.91 × 10^−4^ were considered to be significant (*α*=0.05/128 GVs).

## Results

**Figure 1:**
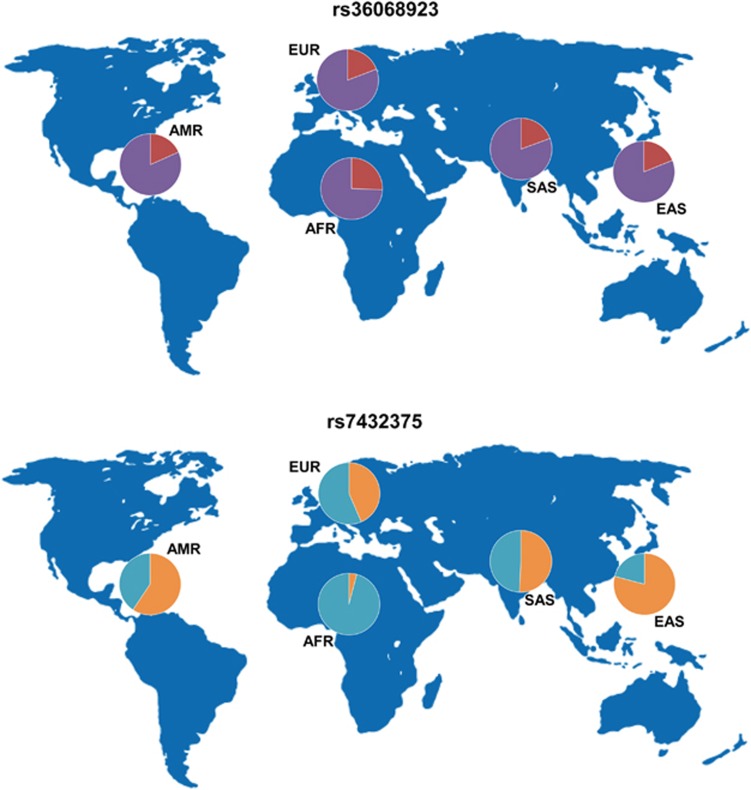
World map of the GVs with the lowest (upper figure) or highest (lower figure) variability across the five ethnic populations. Each pie is proportional to the allele frequency of the GV in each ethnic population. AFR, African; AMR, American; EAS, East Asian; EUR, European; GV, genetic variant; SAS, South Asian.

**Figure 2:**
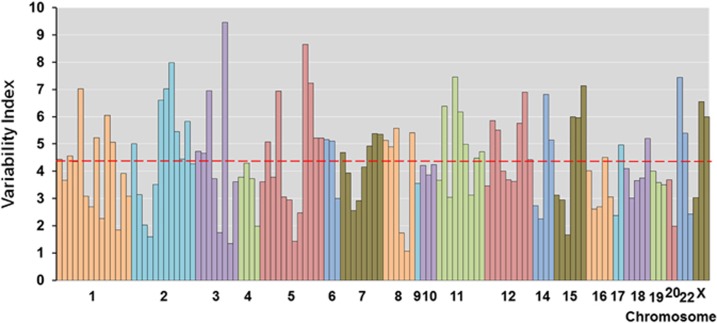
The VI of each GV among the chromosomes. The dotted red line represents the mean VI of the total GVs. As no GVs on chromosomes 13 and 21 have been reached genome-wide significance in the original GWAS,^7^ any GVs on chromosomes 13 and 21 were not listed in this figure. GV, genetic variant; GWAS, genome-wide association study; VI, variable indices.

First, we investigated the genetic variability of 128 independent schizophrenia-associated variants among five major ethnic populations by using the VI. Exactly 122 of the 128 GVs were found in the 1000GP Phase 3 Browser. As shown in Supplementary Figure 1, principal component analysis of the allele frequencies of the 122 GVs shared among the five ethnic populations reflected the populations' structure. The VIs of these GVs ranged from 1.07 to 9.46 (Supplementary Table 2). The top 10 GVs in low or high variability are shown in Table 1. The GV with the lowest variability was rs36068923 on chromosome 8 (Figure 1, VI=1.07). The minor G-allele frequencies at rs36068923 within each ethnic population were 0.189, 0.192, 0.256, 0.183 and 0.194 in the EAS, EUR, AFR, AMR and SAS populations, respectively. In contrast, the GV with the highest variability was rs7432375 on chromosome 3 (Figure 1, VI=9.46). The minor A-allele frequencies at rs36068923 were 0.791, 0.435, 0.041, 0.594 and 0.508 in the EAS, EUR, AFR, AMR and SAS populations, respectively. In addition, 14 GVs had MAFs<0.01 in the EAS population (rs77149735, rs79212538, rs117074560, rs76869799, chr2_149429178_D, rs12826178, rs72934570, rs35518360, rs73229090, rs78322266, rs140505938, rs17194490, rs12522290 and rs111294930), 14 GVs had MAFs<0.01 in the AFR population (rs78322266, rs76869799, rs79212538, rs140505938, rs73229090, rs35518360, rs77149735, rs117074560, rs12826178, chr2_149429178_D, rs1378559, chr7_24747494_D, rs55833108 and rs75059851) and 4 GVs had MAFs<0.01 in the SAS population (rs78322266, rs77149735, rs79212538 and rs35518360). No GVs had MAFs<0.01 in the EUR or AMR populations. The MAFs of four SNPs (rs78322266, rs77149735, rs79212538 and rs35518360) were <0.01 in the EAS, AFR and SAS populations. We also investigated whether the mean genetic variability of each chromosome differed among the chromosomes. As shown in Figure 2, the mean genetic variability did not differ among the chromosomes (F_20, 101_=0.50, *P*=0.96). The mean VIs of the total GVs and highest and lowest chromosomes were 4.34±1.66 (*n*=122); the variability was 5.19±1.89 on chromosome X (*n*=3) and 2.84±1.20 on chromosome 20 (*n*=2). In addition, according to the GWAS rank of each GV (Supplementary Table 2), the schizophrenia-associated 128 GVs were divided into four groups to compare the mean ranks of the VI among groups, where high rank represents low genetic variability. The mean ranks of the VI did not differ among four groups (first group (GWAS top 1–25% ranked GVs): mean ranks of the VI±s.d.=73.3±30.6, second (26–50%): 58.8±39.6, third (51–75%): 50.0±34.2, fourth (76–100%): 65.0±34.0, *z*=7.10, *P*=0.069), suggesting that the VI of each GV is not associated with its significance with schizophrenia in GWAS PGC-II.

**Figure 3:**
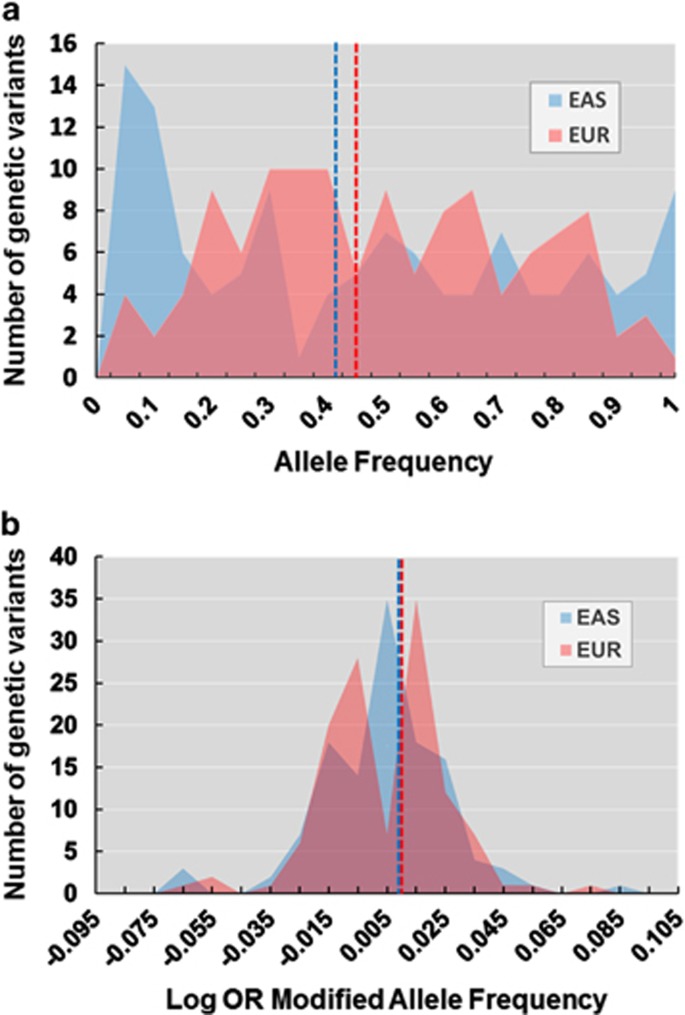
The distributions of the allele frequency at each GV between the EAS and EUR populations. The dotted lines represent the average allele frequency of the GVs in the EAS (blue) and EUR (red) populations. EAS, East Asian; EUR, European; GV, genetic variant; OR, odds ratio.

Next, we focused on the genetic variability of the schizophrenia-associated variants between the EAS and EUR populations utilized in our first analysis because these groups represented the major ethnicities that participated in a previous GWAS.^7^ The allele frequencies of 86 GVs significantly differed between the EAS and EUR populations (*P*<3.91 × 10^−4^; Supplementary Table 3). The top 10 GVs in high or low variability between the EAS and EUR groups are shown in Table 2. The GV with the highest variability was rs4330281 on chromosome 3 (odds ratio (OR)=0.02, 95% confidence intervals=0.01–0.03, *P*=1.55 × 10^−138^). The T-allele frequencies were 0.023 and 0.519 in the EAS and EUR populations, respectively. The GV with the lowest variability was rs2332700 on chromosome 14 (OR=1.00, 95% confidence intervals=0.81–1.23, *P=*9.80 × 10^−1^). The C-allele frequency was similar between the two populations, 0.246 and 0.247 in the EAS and EUR populations, respectively. Compared with randomly selected 128 GVs (Index SNP+1 Mb, MAF>1% of total samples) from genomic regions 1 Mb away from the schizophrenia-associated 128 GVs (68/128 GVs, 53.1%), the schizophrenia-associated GVs showed marginally higher genetic variability between the EAS and EUR populations (86/122 GVs, 70.5%*; χ*^2^=7.25, *P*=7.10 × 10^−3^). Interestingly, the mean allele frequencies of 122 GVs did not statistically differed between these populations (Figure 3a, EAS: mean allele frequency±s.d.=0.443±0.325; EUR: 0.468±0.245, *z*=0.934, *P*=0.350). As each GV does not contribute to pathogenesis of schizophrenia with same effect sizes, the mean allele frequencies were calculated by weighing the allele frequency by the logarithm of the OR (log OR) of each variant in the original GWAS.^7^ This finding did not change after modifying the log OR of each variant (Figure 3b, EAS: −0.00087±0.022; EUR: −0.00099±0.020, *z*=0.071, *P*=0.944).

## Discussion

To the best of our knowledge, this study is the first to examine the genetic variability of the 128 LD-independent schizophrenia-associated GVs detected by the GWAS PGC-II^7^ among five major ethnic populations: EAS, EUR, AMR, AFR and SAS. To compare genetic variability among ethnic populations, we calculated a VI. The VIs of the GVs ranged from 1.07 to 9.46. We successfully detected GVs with high or low genetic variability by using the VI. When we focused on the genetic variability between the EAS and EUR populations (the major ethnic groups included in the previous GWAS),^7^ ~70% of the allele frequencies of the schizophrenia-associated GVs significantly differed between these populations. However, the mean allele frequency of the GVs did not differ between these populations. Consistently with the findings of polygenic risk score studies,^16, 17^ our results suggest that the sum of the GVs contributes to the pathogenesis of schizophrenia across ethnic populations.

Several GVs showed genetic heterogeneity across ethnic populations. GVs with MAFs<0.01 were identified in the EAS (*n=*4), AFR (*n=*14) and SAS (*n*=4) populations. Four GVs with MAFs<0.01 were shared by these ethnic groups. Given that the genetic risk for schizophrenia is due to many GVs with small effects, a cumulative GV effect might be associated with the pathogenesis of schizophrenia, rather than each genetic effect individually. However, as shown in Table 1, some GVs such as rs36068923 and chr3_180594593_I had low genetic heterogeneity across the ethnic populations. Genes near these GVs may be better targets for drug discovery because the number of individuals with these risk variants is consistent across populations.

As predicted by the out-of-Africa model of human origin,^18^ AFR had a greater number of GV sites than the other ethnic populations.^15^ Therefore, we excluded AFR individuals and recalculated the VIs of the schizophrenia-associated GVs in the remaining four populations. The VIs of the GVs in these four ethnic groups ranged from 0.83 to 8.22. The mean VI of the total GVs was 3.55. Although each VI and the range of the VIs in the four ethnicities were significantly lower than the VIs in the five ethnic groups (*z*=−3.87, *P*<0.05), genetic heterogeneities were nevertheless observed. Some of these risk GVs may exert as the onset of schizophrenia in a specific environmental backgrounds, such as climate and infection exposures. Given that environmental exposures as well as individual common genetic risk variants confer risk of schizophrenia, gene–environment interactions (G × E) could have an important role in the etiology of schizophrenia.^19^ Further studies are needed to reveal G × E involving these GVs detected in the GWAS PGC-II.

The major histocompatibility complex on chromosome 6 is one of the strongest and most persistently well-replicated regions associated with schizophrenia according to previous GWASs.^7, 8, 9^ Numerous genome-wide significant variants within the major histocompatibility complex region have been identified. However, it is difficult to analyze this region because of its high LD and ethnic heterogeneity.^20, 21^ We hypothesized that the rs115329265 GV in this region would show high genetic variability among the ethnic populations, and GVs on chromosome 6 would have higher genetic variability than those on other chromosomes. As expected, the rs115329265 GV showed high genetic heterogeneity (VI=5.16). The MAFs of this variant were 0.022, 0.151, 0.445, 0.146 and 0.088 in the EAS, EUR, AFR, AMR and SAS populations, respectively. The VI of this variant was the 36th highest of 122 GVs. In contrast, the GVs on chromosome 6 (VI=4.42) did not show significantly higher genetic variability than those on other chromosomes (VI=4.34). Furthermore, no specific chromosome with high genetic variability was identified among the ethnic populations.

For the majority of these 108 loci, the molecular mechanisms that underlie susceptibility to schizophrenia are unknown. Although 75% of the 108 loci harbor protein-coding genes and 40% harbor a single gene,^7^ most associated variants were not in LD with known protein-coding variants, splice sites or 3’/5’ untranslated regions. In general, SNPs associated with common diseases and phenotypes identified by previous GWASs are enriched in regulatory regions of the genome.^22, 23^ These findings suggest that most GWAS-detected SNPs contribute to disease susceptibility by altering gene expression rather than the protein structure. Therefore, careful examinations of gene expression and its relationship to GVs have become a critical step in elucidating the genetic basis of schizophrenia.^24, 25, 26, 27, 28^

The current study sought to identify genetic variability in schizophrenia-associated GVs detected by a previous GWAS (PGC-II) among five major ethnic populations. As expected, numerous GVs showed genetic heterogeneities among these populations. In particular, 86 of 122 GVs showed significant genetic heterogeneities between the EAS and EUR populations. However, a composite of these GVs did not differ between these populations. Our findings suggest that the cumulative effect of GVs contributes to the risk of schizophrenia across ethnic populations.

## Figures and Tables

**Table 1:** GVs with the lowest or highest variability in the five ethnic populations

*GWAS rank*	*Index SNP*	*Chr*		*MAF*					*Variability index*
				*EAS*	*EUR*	*AFR*	*AMR*	*SAS*	
*GVs with the lowest variability*									
35	rs36068923	8	G	0.189	0.192	0.256	0.183	0.194	1.07
28	chr3_180594593_I	3	I	0.170	0.218	0.316	0.183	0.257	1.34
128	chr5_140143664_I	5	I	0.473	0.467	0.689	0.435	0.481	1.43
62	chr2_146436222_I	2	I	0.095	0.185	0.163	0.101	0.096	1.60
97	rs12148337	15	T	0.534	0.482	0.729	0.483	0.529	1.67
88	rs7819570	8	T	0.103	0.199	0.261	0.120	0.197	1.73
93	rs832187	3	C	0.532	0.402	0.676	0.370	0.370	1.75
68	rs77149735	1	A	0	0.022	0.004	0.010	0.003	1.85
84	rs1106568	4	G	0.267	0.250	0.341	0.206	0.416	1.99
122	rs7267348	20	C	0.238	0.267	0.388	0.291	0.257	1.99

*GVs with the highest variability*									
42	rs7432375	3	A	0.791	0.435	0.041	0.594	0.508	9.46
44	rs111294930	5	G	0.004	0.281	0.013	0.285	0.300	8.65
83	rs59979824	2	A	0.412	0.336	0.023	0.458	0.242	7.98
114	rs12421382	11	T	0.232	0.344	0.017	0.398	0.206	7.45
34	rs9607782	22	A	0.056	0.265	0.152	0.497	0.327	7.44
45	rs2973155	5	T	0.596	0.359	0.079	0.460	0.493	7.23
11	rs4702	15	G	0.475	0.437	0.059	0.411	0.501	7.14
22	rs12129573	1	A	0.203	0.397	0.108	0.542	0.321	7.03
18	rs11693094	2	T	0.500	0.422	0.061	0.429	0.404	7.02
12	rs75968099	3	T	0.055	0.363	0.060	0.323	0.321	6.96

Abbreviations: AFR, African; AMR, American; Chr, chromosome; EAS, East Asian; EUR, European; GV, genetic variant; GWAS, genome-wide association study; I, insertion; MAF, minor allele frequency; SAS, South Asian; SNP, single-nucleotide polymorphism.

**Table 2:** High and low genetic variability between the EAS and EUR populations

*GWAS rank*	*Index SNP*	*Chr*	*A12*	*GWAS PGC-II*				*Population genetics*			
				*Frq*_*case*_	*Frq*_*control*_	*OR (95% CIs)*	P	*Frq*_*EAS*_	*Frq*_*EUR*_	*OR (95% CIs)*	P
*High genetic variability*											
72	rs4330281	3	TC	0.479	0.48	0.94 (0.92–0.96)	**4.64 × 10**^**−9**^	0.023	0.519	0.02 (0.01–0.03)	**1.55 × 10**^**−138**^
96	rs8082590	17	AG	0.611	0.614	0.94 (0.92–0.96)	**1.77 × 10**^**−8**^	0.095	0.623	0.06 (0.05–0.08)	**1.10 × 10**^**−134**^
74	rs2693698	14	AG	0.412	0.418	0.94 (0.92–0.96)	**4.80 × 10**^**−9**^	0.032	0.466	0.04 (0.03–0.05)	**1.31 × 10**^**−112**^
58	rs4766428	12	TC	0.481	0.474	1.07 (1.05–1.09)	**1.40 × 10**^**−9**^	0.917	0.453	13.25 (10.22–17.33)	**5.14 × 10**^**−111**^
15	rs8042374	15	AG	0.75	0.725	1.09 (1.07–1.12)	**2.44 × 10**^**−13**^	0.267	0.759	0.12 (0.09–0.14)	**2.36 × 10**^**−108**^
110	rs4388249	5	TC	0.212	0.213	1.08 (1.05–1.10)	**3.05 × 10**^**−8**^	0.622	0.154	9.02 (7.26–11.26)	**5.89 × 10**^**−103**^
112	rs11740474	5	AT	0.601	0.621	0.94 (0.92–0.96)	**3.15 × 10**^**−8**^	0.952	0.588	13.96 (10.15–19.59)	**5.05 × 10**^**−84**^
37	rs2514218	11	TC	0.31	0.314	0.93 (0.91–0.95)	**2.75 × 10**^**−11**^	0.031	0.352	0.06 (0.04–0.09)	**5.18 × 10**^**−75**^
44	rs111294930	5	AG	0.788	0.782	1.09 (1.06–1.12)	**1.06 × 10**^**−10**^	0.996	0.719	98.03 (37.64–363.52)	**6.71 × 10**^**−71**^
12	rs75968099	3	TC	0.346	0.324	1.09 (1.06–1.11)	**1.05 × 10**^**−13**^	0.055	0.363	0.10 (0.07–0.14)	**5.29 × 10**^**−65**^

*Low genetic variability*											
75	rs2332700	14	CG	0.262	0.249	1.07 (1.05–1.10)	**4.86 × 10**^**−9**^	0.246	0.247	1.00 (0.81–1.23)	9.80 × 10^−1^
35	rs36068923	8	AG	0.787	0.803	0.92 (0.90–0.94)	**2.61 × 10**^**−11**^	0.811	0.808	1.02 (0.81–1.28)	8.93 × 10^−1^
17	rs10791097	11	TG	0.479	0.46	1.08 (1.06–1.10)	**1.09 × 10**^**-12**^	0.477	0.474	1.01 (0.85–1.21)	8.92 × 10^−1^
98	rs12325245	16	AT	0.849	0.859	0.92 (0.89–0.95)	**1.87 × 10**^**-8**^	0.851	0.847	1.03 (0.80–1.33)	7.89 × 10^−1^
128	chr5_140143664_I	5	I12D	0.486	0.475	1.06 (1.04–1.08)	**4.85 × 10**^**-8**^	0.473	0.467	1.02 (0.86–1.23)	7.87 × 10^−^^1^
9	rs2851447	12	CG	0.723	0.741	0.92 (0.89–0.94)	**1.86 × 10**^**-14**^	0.734	0.724	1.05 (0.86–1.29)	5.97 × 10^−^^1^
120	rs6670165	1	TC	0.196	0.184	1.08 (1.05–1.10)	**4.45 × 10**^**-8**^	0.19	0.201	0.94 (0.75–1.17)	5.59 × 10^−^^1^
65	rs1498232	1	TC	0.311	0.296	1.07 (1.05–1.09)	**2.86 × 10**^**-9**^	0.295	0.307	0.94 (0.78–1.15)	5.40 × 10^−^^1^
77	rs6984242	8	AG	0.586	0.6	0.94 (0.92–0.96)	**5.97 × 10**^**-9**^	0.617	0.603	1.06 (0.88–1.27)	5.29 × 10^−1^
84	rs1106568	4	AG	0.747	0.761	0.93 (0.91–0.96)	**9.47 × 10**^**-9**^	0.733	0.75	0.91 (0.74–1.12)	3.73 × 10^−1^

Abbreviations: Chr, chromosome; CI, confidence interval; D, deletion; EAS, East Asian; EUR, European; Frq, frequency; GWAS, genome-wide association study; I, insertion; OR, odds ratio; PGC, Psychiatric Genomics Consortium; SNP, single-nucleotide polymorphism.

Significant *P*-values are shown in boldface and underlined.
