# Supplementary material for: Variability of 128 schizophrenia-associated gene variants across distinct ethnic populations
Source: Transl Psychiatry. 2017 Jan 3;7(1):e988–. doi: 10.1038/tp.2016.260 (PMC5545726; doi:10.1038/tp.2016.260)
Supplement: Supplementary Information [file tp2016260x1.doc]

**Supplementary Information**

**Supplementary Figure 1.** A principal component analysis of the allele frequencies of 122 GVs in the five ethnic populations. Scatterplots of principal components 1 (PC1) and 2 (PC2) are shown. EAS: East Asian, EUR: European, AFR: African, AMR: American, SAS: South Asian

**Supplementary Table 1. Demographic information for the participants in this study**

| **Super Population Code** | **Population**  **Code** | **Population Description** | ***n*** | **Female/Male**  **(%Female)** |
| --- | --- | --- | --- | --- |
| EAS | CHB | Han Chinese in Bejing, China | 103 | 57/46 (55.3) |
| EAS | JPT | Japanese in Tokyo, Japan | 104 | 48/56 (46.2) |
| EAS | CHS | Southern Han Chinese | 105 | 53/52 (50.5) |
| EAS | CDX | Chinese Dai in Xishuangbanna, China | 93 | 49/44 (52.7) |
| EAS | KHV | Kinh in Ho Chi Minh City, Vietnam | 99 | 53/46 (53.5) |
| EUR | CEU | Utah Residents (CEPH) with Northern and Western Ancestry | 99 | 50/49 (50.5) |
| EUR | TSI | Toscani in Italia | 107 | 54/53 (50.5) |
| EUR | FIN | Finnish in Finland | 99 | 61/38 (61.6) |
| EUR | GBR | British in England and Scotland | 91 | 45/46 (49.5) |
| EUR | IBS | Iberian Population in Spain | 107 | 53/54 (49.5) |
| AFR | YRI | Yoruba in Ibadan, Nigeria | 108 | 56/52 (51.9) |
| AFR | LWK | Luhya in Webuye, Kenya | 99 | 55/44 (55.6) |
| AFR | GWD | Gambian in Western Divisions in the Gambia | 113 | 58/55 (51.3) |
| AFR | MSL | Mende in Sierra Leone | 85 | 43/42 (50.6) |
| AFR | ESN | Esan in Nigeria | 99 | 46/53 (46.5) |
| AFR | ASW | Americans of African Ancestry in SW USA | 61 | 35/26 (57.4) |
| AFR | ACB | African Caribbeans in Barbados | 96 | 49/47 (51.0) |
| AMR | MXL | Mexican Ancestry from Los Angeles USA | 64 | 32/32 (50.0) |
| AMR | PUR | Puerto Ricans from Puerto Rico | 104 | 50/54 (48.1) |
| AMR | CLM | Colombians from Medellin, Colombia | 94 | 51/43 (54.3) |
| AMR | PEL | Peruvians from Lima, Peru | 85 | 44/41 (51.8) |
| SAS | GIH | Gujarati Indian from Houston, Texas | 103 | 47/56 (45.6) |
| SAS | PJL | Punjabi from Lahore, Pakistan | 96 | 48/48 (50.0) |
| SAS | BEB | Bengali from Bangladesh | 86 | 44/42 (51.2) |
| SAS | STU | Sri Lankan Tamil from the UK | 102 | 47/55 (46.1) |
| SAS | ITU | Indian Telugu from the UK | 102 | 43/59 (42.2) |

EAS: East Asian, EUR: European, AFR: African, AMR: American, SAS: South Asian. All donors were over 18 and declared themselves to be healthy at the time of collection.

**Supplementary Table 2. The variability of 128 GVs in five ethnic populations**

| GWAS |  |  | MAF | | | | | | Variability |
| --- | --- | --- | --- | --- | --- | --- | --- | --- | --- |
| Rank | Index SNP | Chr |  | EAS | EUR | AFR | AMR | SAS | Index |
| 1 | rs115329265 | 6 | G | 0.022 | 0.151 | 0.445 | 0.146 | 0.088 | 5.16 |
| 2 | rs1702294 | 1 | T | 0.070 | 0.210 | 0.172 | 0.107 | 0.223 | 2.70 |
| 3 | rs11191419 | 10 | A | 0.588 | 0.352 | 0.314 | 0.402 | 0.322 | 3.87 |
| 4 | rs2007044 | 12 | G | 0.327 | 0.366 | 0.828 | 0.448 | 0.391 | 3.46 |
| 5 | rs4129585 | 8 | A | 0.264 | 0.445 | 0.069 | 0.269 | 0.209 | 5.41 |
| 6 | rs35518360 | 4 | T | 0.000 | 0.092 | 0.003 | 0.049 | 0.006 | 4.30 |
| 7 | chr7_2025096_I | 7 | D | 0.547 | 0.420 | 0.388 | 0.610 | 0.421 | 4.69 |
| 8 | rs4391122 | 5 | G | 0.218 | 0.469 | 0.896 | 0.539 | 0.511 | 5.08 |
| 9 | rs2851447 | 12 | G | 0.266 | 0.276 | 0.887 | 0.330 | 0.248 | 4.42 |
| 10 | chr2_200825237_I | 2 | D | NA | NA | NA | NA | NA | - |
| 11 | rs4702 | 15 | G | 0.475 | 0.437 | 0.059 | 0.411 | 0.501 | 7.14 |
| 12 | rs75968099 | 3 | T | 0.055 | 0.363 | 0.060 | 0.323 | 0.321 | 6.96 |
| 13 | chr10_104957618_I | 10 | I | NA | NA | NA | NA | NA | - |
| 14 | rs12887734 | 14 | T | 0.352 | 0.274 | 0.125 | 0.336 | 0.120 | 5.15 |
| 15 | rs8042374 | 15 | G | 0.733 | 0.241 | 0.324 | 0.549 | 0.471 | 6.00 |
| 16 | rs13240464 | 7 | C | 0.530 | 0.375 | 0.239 | 0.418 | 0.639 | 4.92 |
| 17 | rs10791097 | 11 | T | 0.477 | 0.474 | 0.207 | 0.405 | 0.346 | 4.48 |
| 18 | rs11693094 | 2 | T | 0.500 | 0.422 | 0.061 | 0.429 | 0.404 | 7.02 |
| 19 | rs1378559 | X | C | 0.305 | 0.180 | 0.007 | 0.044 | 0.077 | 6.55 |
| 20 | rs7893279 | 10 | G | 0.086 | 0.107 | 0.039 | 0.202 | 0.089 | 4.21 |
| 21 | rs12826178 | 12 | T | 0.000 | 0.075 | 0.004 | 0.036 | 0.035 | 3.68 |
| 22 | rs12129573 | 1 | A | 0.203 | 0.397 | 0.108 | 0.542 | 0.321 | 7.03 |
| 23 | rs6704768 | 2 | G | 0.537 | 0.471 | 0.486 | 0.550 | 0.279 | 4.27 |
| 24 | rs55661361 | 11 | A | 0.298 | 0.351 | 0.752 | 0.210 | 0.244 | 3.12 |
| 25 | rs9636107 | 18 | G | 0.801 | 0.490 | 0.514 | 0.503 | 0.702 | 3.65 |
| 26 | chr11_46350213_D | 11 | D | 0.085 | 0.154 | 0.871 | 0.135 | 0.148 | 6.38 |
| 27 | rs7907645 | 10 | G | NA | NA | NA | NA | NA | - |
| 28 | chr3_180594593_I | 3 | I | 0.170 | 0.218 | 0.316 | 0.183 | 0.257 | 1.34 |
| 29 | rs6065094 | 20 | A | 0.304 | 0.353 | 0.200 | 0.354 | 0.287 | 3.68 |
| 30 | rs11682175 | 2 | C | 0.361 | 0.446 | 0.179 | 0.447 | 0.406 | 5.00 |
| 31 | rs950169 | 15 | T | 0.054 | 0.273 | 0.011 | 0.179 | 0.182 | 5.96 |
| 32 | rs72934570 | 18 | T | 0.000 | 0.082 | 0.014 | 0.045 | 0.012 | 3.75 |
| 33 | rs6434928 | 2 | G | 0.482 | 0.274 | 0.118 | 0.369 | 0.244 | 5.45 |
| 34 | rs9607782 | 22 | A | 0.056 | 0.265 | 0.152 | 0.497 | 0.327 | 7.44 |
| 35 | rs36068923 | 8 | G | 0.189 | 0.192 | 0.256 | 0.183 | 0.194 | 1.07 |
| 36 | rs17194490 | 3 | T | 0.003 | 0.160 | 0.036 | 0.104 | 0.094 | 4.72 |
| 37 | rs2514218 | 11 | T | 0.031 | 0.352 | 0.120 | 0.285 | 0.212 | 6.17 |
| 38 | rs75059851 | 11 | G | 0.053 | 0.201 | 0.009 | 0.120 | 0.109 | 4.71 |
| 39 | rs2535627 | 3 | C | 0.431 | 0.500 | 0.705 | 0.601 | 0.395 | 3.73 |
| 40 | rs12691307 | 16 | G | 0.379 | 0.522 | 0.414 | 0.396 | 0.526 | 2.69 |
| 41 | chr22_39987017_D | 22 | D | NA | NA | NA | NA | NA | - |
| 42 | rs7432375 | 3 | A | 0.791 | 0.435 | 0.041 | 0.594 | 0.508 | 9.46 |
| 43 | chr18_52749216_D | 18 | D | 0.599 | 0.386 | 0.316 | 0.412 | 0.287 | 4.10 |
| 44 | rs111294930 | 5 | G | 0.004 | 0.281 | 0.013 | 0.285 | 0.300 | 8.65 |
| 45 | rs2973155 | 5 | T | 0.596 | 0.359 | 0.079 | 0.460 | 0.493 | 7.23 |
| 46 | rs5937157 | X | G | 0.120 | 0.249 | 0.443 | 0.212 | 0.033 | 6.00 |
| 47 | rs4523957 | 17 | G | 0.321 | 0.385 | 0.716 | 0.367 | 0.487 | 2.38 |
| 48 | rs12704290 | 7 | A | 0.081 | 0.128 | 0.024 | 0.056 | 0.075 | 2.56 |
| 49 | rs12903146 | 15 | G | 0.718 | 0.431 | 0.762 | 0.552 | 0.535 | 2.94 |
| 50 | rs11210892 | 1 | G | 0.314 | 0.362 | 0.902 | 0.527 | 0.549 | 4.35 |
| 51 | rs2905426 | 19 | G | 0.412 | 0.331 | 0.933 | 0.499 | 0.642 | 4.01 |
| 52 | rs140505938 | 1 | T | 0.001 | 0.169 | 0.002 | 0.062 | 0.027 | 5.23 |
| 53 | chr6_84280274_D | 6 | D | NA | NA | NA | NA | NA | - |
| 54 | rs4648845 | 1 | C | 0.832 | 0.476 | 0.532 | 0.644 | 0.669 | 4.44 |
| 55 | rs7405404 | 16 | T | 0.117 | 0.260 | 0.185 | 0.193 | 0.162 | 2.61 |
| 56 | rs6466055 | 7 | A | 0.297 | 0.356 | 0.595 | 0.343 | 0.222 | 2.92 |
| 57 | chr1_8424984_D | 1 | D | 0.082 | 0.320 | 0.256 | 0.219 | 0.253 | 3.67 |
| 58 | rs4766428 | 12 | T | 0.917 | 0.453 | 0.189 | 0.382 | 0.700 | 6.90 |
| 59 | rs10520163 | 4 | T | 0.253 | 0.496 | 0.825 | 0.340 | 0.609 | 3.72 |
| 60 | rs117074560 | 6 | T | 0.000 | 0.038 | 0.004 | 0.032 | 0.030 | 3.00 |
| 61 | rs6002655 | 22 | T | 0.662 | 0.425 | 0.657 | 0.484 | 0.508 | 2.43 |
| 62 | chr2_146436222_I | 2 | I | 0.095 | 0.185 | 0.163 | 0.101 | 0.096 | 1.60 |
| 63 | rs9420 | 11 | A | 0.097 | 0.302 | 0.341 | 0.177 | 0.271 | 3.04 |
| 64 | rs11027857 | 11 | G | 0.649 | 0.438 | 0.471 | 0.549 | 0.574 | 3.67 |
| 65 | rs1498232 | 1 | T | 0.295 | 0.307 | 0.887 | 0.484 | 0.569 | 4.56 |
| 66 | rs3735025 | 7 | C | 0.321 | 0.360 | 0.093 | 0.363 | 0.338 | 5.36 |
| 67 | rs11139497 | 9 | A | 0.674 | 0.338 | 0.873 | 0.362 | 0.355 | 3.56 |
| 68 | rs77149735 | 1 | A | 0.000 | 0.022 | 0.004 | 0.010 | 0.003 | 1.85 |
| 69 | rs56205728 | 15 | A | 0.120 | 0.288 | 0.238 | 0.222 | 0.131 | 3.13 |
| 70 | rs2053079 | 19 | G | 0.180 | 0.259 | 0.082 | 0.197 | 0.220 | 3.58 |
| 71 | rs16867576 | 5 | G | 0.031 | 0.110 | 0.344 | 0.097 | 0.105 | 3.78 |
| 72 | rs4330281 | 3 | T | 0.977 | 0.481 | 0.570 | 0.624 | 0.628 | 4.66 |
| 73 | rs3849046 | 5 | C | 0.785 | 0.491 | 0.548 | 0.401 | 0.641 | 2.95 |
| 74 | rs2693698 | 14 | A | 0.032 | 0.466 | 0.435 | 0.327 | 0.157 | 6.81 |
| 75 | rs2332700 | 14 | C | 0.246 | 0.247 | 0.232 | 0.242 | 0.170 | 2.25 |
| 76 | rs1501357 | 5 | C | 0.462 | 0.184 | 0.635 | 0.316 | 0.297 | 3.61 |
| 77 | rs6984242 | 8 | G | 0.383 | 0.397 | 0.127 | 0.272 | 0.620 | 5.58 |
| 78 | chr1_243881945_I | 1 | D | 0.575 | 0.344 | 0.933 | 0.447 | 0.550 | 3.09 |
| 79 | rs79212538 | 5 | T | 0.000 | 0.036 | 0.001 | 0.012 | 0.003 | 2.47 |
| 80 | rs3768644 | 2 | A | 0.034 | 0.116 | 0.134 | 0.052 | 0.050 | 2.03 |
| 81 | rs77502336 | 11 | C | 0.437 | 0.300 | 0.132 | 0.333 | 0.453 | 4.99 |
| 82 | rs6704641 | 2 | G | 0.464 | 0.169 | 0.528 | 0.278 | 0.140 | 4.45 |
| 83 | rs59979824 | 2 | A | 0.412 | 0.336 | 0.023 | 0.458 | 0.242 | 7.98 |
| 84 | rs1106568 | 4 | G | 0.267 | 0.250 | 0.341 | 0.206 | 0.416 | 1.99 |
| 85 | rs10503253 | 8 | A | 0.352 | 0.217 | 0.077 | 0.098 | 0.403 | 5.13 |
| 86 | rs10043984 | 5 | T | 0.141 | 0.261 | 0.274 | 0.226 | 0.109 | 3.06 |
| 87 | rs11685299 | 2 | A | 0.364 | 0.330 | 0.061 | 0.344 | 0.257 | 5.82 |
| 88 | rs7819570 | 8 | T | 0.103 | 0.199 | 0.261 | 0.120 | 0.197 | 1.73 |
| 89 | rs715170 | 18 | T | 0.165 | 0.286 | 0.023 | 0.182 | 0.221 | 5.20 |
| 90 | rs9922678 | 16 | A | 0.123 | 0.321 | 0.630 | 0.255 | 0.254 | 4.02 |
| 91 | rs78322266 | 18 | T | 0.001 | 0.033 | 0.000 | 0.024 | 0.000 | 3.01 |
| 92 | rs2068012 | 14 | C | 0.244 | 0.220 | 0.348 | 0.321 | 0.340 | 2.73 |
| 93 | rs832187 | 3 | C | 0.532 | 0.402 | 0.676 | 0.370 | 0.370 | 1.75 |
| 94 | rs8044995 | 16 | A | 0.099 | 0.161 | 0.374 | 0.207 | 0.203 | 3.05 |
| 95 | chr2_149429178_D | 2 | D | 0.000 | 0.053 | 0.006 | 0.050 | 0.026 | 3.52 |
| 96 | rs8082590 | 17 | G | 0.905 | 0.377 | 0.530 | 0.533 | 0.778 | 4.97 |
| 97 | rs12148337 | 15 | T | 0.534 | 0.482 | 0.729 | 0.483 | 0.529 | 1.67 |
| 98 | rs12325245 | 16 | T | 0.149 | 0.153 | 0.113 | 0.278 | 0.321 | 4.50 |
| 99 | rs2239063 | 12 | C | 0.263 | 0.301 | 0.046 | 0.316 | 0.325 | 5.86 |
| 100 | rs12522290 | 5 | G | 0.003 | 0.183 | 0.035 | 0.108 | 0.140 | 5.21 |
| 101 | rs10803138 | 1 | A | 0.028 | 0.267 | 0.235 | 0.151 | 0.339 | 5.06 |
| 102 | rs73229090 | 8 | A | 0.000 | 0.113 | 0.002 | 0.069 | 0.040 | 4.89 |
| 103 | rs324017 | 12 | A | 0.464 | 0.283 | 0.174 | 0.269 | 0.405 | 4.01 |
| 104 | rs12845396 | X | T | 0.297 | 0.234 | 0.457 | 0.334 | 0.247 | 3.03 |
| 105 | rs55833108 | 10 | T | 0.082 | 0.187 | 0.009 | 0.102 | 0.074 | 4.24 |
| 106 | rs9841616 | 3 | A | 0.133 | 0.179 | 0.520 | 0.254 | 0.246 | 3.61 |
| 107 | rs76869799 | 1 | G | 0.000 | 0.042 | 0.000 | 0.023 | 0.015 | 3.09 |
| 108 | rs1339227 | 6 | T | 0.401 | 0.342 | 0.080 | 0.225 | 0.379 | 5.10 |
| 109 | chr7_24747494_D | 7 | D | 0.056 | 0.101 | 0.007 | 0.117 | 0.078 | 3.93 |
| 110 | rs4388249 | 5 | T | 0.622 | 0.154 | 0.064 | 0.244 | 0.382 | 6.94 |
| 111 | rs215411 | 4 | A | 0.071 | 0.305 | 0.393 | 0.189 | 0.222 | 3.78 |
| 112 | rs11740474 | 5 | T | 0.048 | 0.412 | 0.348 | 0.233 | 0.311 | 5.22 |
| 113 | rs1023500 | 22 | C | 0.123 | 0.201 | 0.401 | 0.450 | 0.326 | 5.40 |
| 114 | rs12421382 | 11 | T | 0.232 | 0.344 | 0.017 | 0.398 | 0.206 | 7.45 |
| 115 | rs211829 | 7 | C | 0.156 | 0.423 | 0.364 | 0.349 | 0.204 | 4.15 |
| 116 | rs679087 | 12 | A | 0.281 | 0.353 | 0.029 | 0.171 | 0.148 | 5.50 |
| 117 | rs75575209 | 2 | T | 0.020 | 0.084 | 0.147 | 0.089 | 0.025 | 3.14 |
| 118 | rs7801375 | 7 | A | 0.040 | 0.153 | 0.581 | 0.140 | 0.261 | 5.38 |
| 119 | rs14403 | 1 | T | 0.257 | 0.207 | 0.054 | 0.166 | 0.201 | 3.92 |
| 120 | rs6670165 | 1 | T | 0.190 | 0.201 | 0.147 | 0.193 | 0.227 | 2.27 |
| 121 | rs7523273 | 1 | G | 0.094 | 0.330 | 0.231 | 0.458 | 0.224 | 6.05 |
| 122 | rs7267348 | 20 | C | 0.238 | 0.267 | 0.388 | 0.291 | 0.257 | 1.99 |
| 123 | rs4240748 | 12 | C | 0.222 | 0.374 | 0.779 | 0.274 | 0.504 | 3.63 |
| 124 | rs2909457 | 2 | G | 0.107 | 0.417 | 0.209 | 0.487 | 0.429 | 6.61 |
| 125 | rs56873913 | 19 | G | 0.193 | 0.218 | 0.518 | 0.291 | 0.448 | 3.50 |
| 126 | rs190065944 | 15 | A | NA | NA | NA | NA | NA | - |
| 127 | rs10860964 | 12 | C | 0.155 | 0.379 | 0.066 | 0.298 | 0.337 | 5.75 |
| 128 | chr5_140143664_I | 5 | I | 0.473 | 0.467 | 0.689 | 0.435 | 0.481 | 1.43 |

GWAS: genome-wide association study, MAF: minor allele frequency, Chr: chromosome, EAS: East Asian, EUR: European, AFR: African, AMR: American, SAS: South Asian, I: insertion, D: deletion

**Supplementary Table 3. The genetic variability of 128 GVs between the EAS and EUR populations**

| GWAS |  |  |  | GWAS PGC-II | | | |  | Population Genetics | | | |
| --- | --- | --- | --- | --- | --- | --- | --- | --- | --- | --- | --- | --- |
| Rank | Index SNP | Chr | A12 | Frq_case_ | Frq_control_ | OR (95% CIs) | *P* |  | Frq _EAS_ | Frq _EUR_ | OR (95% CIs) | *P* |
| 1 | rs115329265 | 6 | AG | 0.864 | 0.850 | 1.21 (1.17-1.24) | **3.48×10^-31^** |  | 0.978 | 0.849 | 7.97 (5.02-13.22) | **5.46×10^-25^** |
| 2 | rs1702294 | 1 | TC | 0.175 | 0.191 | 0.89 (0.87-0.91) | **3.36×10^-19^** |  | 0.070 | 0.210 | 0.29 (0.21-0.38) | **2.10×10^-19^** |
| 3 | rs11191419 | 10 | AT | 0.337 | 0.360 | 0.91 (0.87-0.93) | **6.20×10^-19^** |  | 0.588 | 0.352 | 2.63 (2.19-3.16) | **2.20×10^-26^** |
| 4 | rs2007044 | 12 | AG | 0.602 | 0.624 | 0.91 (0.89-0.93) | **3.22×10^-18^** |  | 0.673 | 0.634 | 1.18 (0.98-1.43) | 7.00×10^-2^ |
| 5 | rs4129585 | 8 | AC | 0.447 | 0.424 | 1.09 (1.07-1.11) | **1.74×10^-15^** |  | 0.264 | 0.445 | 0.38 (0.31-0.46) | **1.73×10^-17^** |
| 6 | rs35518360 | 4 | AT | 0.909 | 0.922 | 0.86 (0.82-0.89) | **7.98×10^-15^** |  | 1.000 | 0.908 | Inf (27.14-Inf) | **4.88×10^-23^** |
| 7 | chr7_2025096_I | 7 | DI3 | 0.405 | 0.423 | 0.92 (0.90-0.94) | **8.20×10^-15^** |  | 0.547 | 0.420 | 1.66 (1.39-1.99) | **1.48×10^-8^** |
| 8 | rs4391122 | 5 | AG | 0.505 | 0.532 | 0.92 (0.90-0.94) | **1.10×10^-14^** |  | 0.782 | 0.531 | 3.16 (2.60-3.86) | **2.00×10^-32^** |
| 9 | rs2851447 | 12 | CG | 0.723 | 0.741 | 0.92 (0.89-0.94) | **1.86×10^-14^** |  | 0.734 | 0.724 | 1.05 (0.86-1.29) | 5.97×10^-1^ |
| 10 | chr2_200825237_I | 2 | I2D | 0.741 | 0.754 | 0.91 (0.89-0.93) | **5.65×10^-14^** |  | NA | NA | NA | NA |
| 11 | rs4702 | 15 | AG | 0.547 | 0.562 | 0.92 (0.90-0.94) | **8.30×10^-14^** |  | 0.525 | 0.563 | 0.86 (0.72-1.03) | 8.84×10^-2^ |
| 12 | rs75968099 | 3 | TC | 0.346 | 0.324 | 1.09 (1.06-1.11) | **1.05×10^-13^** |  | 0.055 | 0.363 | 0.10 (0.07-0.14) | **5.29×10^-65^** |
| 13 | chr10_104957618_I | 10 | I2D | 0.065 | 0.076 | 0.84 (0.81-0.88) | **1.06×10^-13^** |  | NA | NA | NA | NA |
| 14 | rs12887734 | 14 | TG | 0.299 | 0.287 | 1.09 (1.06-1.11) | **1.36×10^-13^** |  | 0.352 | 0.274 | 1.44 (1.18-1.75) | **1.67×10^-4^** |
| 15 | rs8042374 | 15 | AG | 0.750 | 0.725 | 1.09 (1.07-1.12) | **2.44×10^-13^** |  | 0.267 | 0.759 | 0.12 (0.09-0.14) | **2.36×10^-108^** |
| 16 | rs13240464 | 7 | TC | 0.667 | 0.647 | 1.08 (1.06-1.11) | **3.03×10^-13^** |  | 0.470 | 0.625 | 0.53 (0.44-0.64) | **2.78×10^-12^** |
| 17 | rs10791097 | 11 | TG | 0.479 | 0.460 | 1.08 (1.06-1.10) | **1.09×10^-12^** |  | 0.477 | 0.474 | 1.01 (0.85-1.21) | 8.92×10^-1^ |
| 18 | rs11693094 | 2 | TC | 0.440 | 0.458 | 0.93 (0.91-0.95) | **1.53×10^-12^** |  | 0.500 | 0.422 | 1.37 (1.14-1.64) | 4.83.×10^-4^ |
| 19 | rs1378559 | X | TC | 0.848 | 0.831 | 1.09 (1.06-1.12) | **1.61×10^-12^** |  | 0.695 | 0.820 | 0.50 (0.39-0.64) | **1.23×10^-8^** |
| 20 | rs7893279 | 10 | TG | 0.899 | 0.889 | 1.13 (1.09-1.16) | **1.97×10^-12^** |  | 0.914 | 0.893 | 1.27 (0.94-1.73) | 1.10×10^-1^ |
| 21 | rs12826178 | 12 | TG | 0.063 | 0.071 | 0.85 (0.81-0.89) | **2.02×10^-12^** |  | 0 | 0.075 | 0.00 (0.00-0.05) | **1.00×10^-18^** |
| 22 | rs12129573 | 1 | AC | 0.377 | 0.358 | 1.08 (1.06-1.10) | **2.03×10^-12^** |  | 0.203 | 0.397 | 0.39 (0.32-0.48) | **2.98×10^-21^** |
| 23 | rs6704768 | 2 | AG | 0.540 | 0.552 | 0.93 (0.91-0.95) | **2.32×10^-12^** |  | 0.463 | 0.529 | 0.77 (0.64-0.92) | 3.27×10^-3^ |
| 24 | rs55661361 | 11 | AG | 0.319 | 0.335 | 0.93 (0.91-0.95) | **2.80×10^-12^** |  | 0.298 | 0.351 | 0.78 (0.65-0.95) | 1.07×10^-2^ |
| 25 | rs9636107 | 18 | AG | 0.490 | 0.503 | 0.93 (0.91-0.95) | **3.34×10^-12^** |  | 0.199 | 0.510 | 0.24 (0.20-0.29) | **4.60×10^-48^** |
| 26 | chr11_46350213_D | 11 | I2D | 0.835 | 0.850 | 0.91 (0.88-0.93) | **1.26×10^-11^** |  | 0.915 | 0.846 | 1.95 (1.46-2.61) | **2.00×10^-6^** |
| 27 | rs7907645 | 10 | TG | 0.900 | 0.888 | 1.14 (1.10-1.19) | **1.27×10^-11^** |  | NA | NA | NA | NA |
| 28 | chr3_180594593_I | 3 | I2D | 0.196 | 0.208 | 0.91 (0.89-0.94) | **1.30×10^-11^** |  | 0.170 | 0.218 | 0.73 (0.58-0.92) | 6.36×10^-3^ |
| 29 | rs6065094 | 20 | AG | 0.307 | 0.322 | 0.93 (0.91-0.95) | **1.46×10^-11^** |  | 0.304 | 0.353 | 0.80 (0.66-0.97) | 1.85×10^-2^ |
| 30 | rs11682175 | 2 | TC | 0.520 | 0.542 | 0.93 (0.91-0.95) | **1.47×10^-11^** |  | 0.639 | 0.554 | 1.43 (1.19-1.71) | **9.74×10^-5^** |
| 31 | rs950169 | 15 | TC | 0.247 | 0.257 | 0.92 (0.90-0.95) | **1.62×10^-11^** |  | 0.054 | 0.273 | 0.15 (0.11-0.21) | **1.35×10^-40^** |
| 32 | rs72934570 | 18 | TC | 0.070 | 0.080 | 0.87 (0.84-0.91) | **1.97×10^-11^** |  | 0 | 0.082 | 0.00 (0.00-0.04) | **2.15×10^-20^** |
| 33 | rs6434928 | 2 | AG | 0.635 | 0.643 | 0.93 (0.91-0.95) | **2.06×10^-11^** |  | 0.518 | 0.726 | 0.41 (0.34-0.49) | **6.98×10^-22^** |
| 34 | rs9607782 | 22 | AT | 0.250 | 0.232 | 1.09 (1.06-1.11) | **2.07×10^-11^** |  | 0.056 | 0.265 | 0.16 (0.12-0.22) | **1.08×10^-37^** |
| 35 | rs36068923 | 8 | AG | 0.787 | 0.803 | 0.92 (0.90-0.94) | **2.61×10^-11^** |  | 0.811 | 0.808 | 1.02 (0.81-1.28) | 8.93×10^-1^ |
| 36 | rs17194490 | 3 | TG | 0.169 | 0.156 | 1.10 (1.07-1.13) | **2.69×10^-11^** |  | 0.003 | 0.160 | 0.02 (0.00-0.05) | **5.38×10^-38^** |
| 37 | rs2514218 | 11 | TC | 0.310 | 0.314 | 0.93 (0.91-0.95) | **2.75×10^-11^** |  | 0.031 | 0.352 | 0.06 (0.04-0.09) | **5.18×10^-75^** |
| 38 | rs75059851 | 11 | AG | 0.812 | 0.797 | 1.09 (1.06-1.12) | **3.87×10^-11^** |  | 0.947 | 0.799 | 4.52 (3.28-6.34) | **1.51×10^-23^** |
| 39 | rs2535627 | 3 | TC | 0.545 | 0.529 | 1.07 (1.05-1.09) | **4.26×10^-11^** |  | 0.569 | 0.500 | 1.32 (1.11-1.58) | 1.78×10^-3^ |
| 40 | rs12691307 | 16 | AG | 0.524 | 0.510 | 1.07 (1.05-1.10) | **4.55×10^-11^** |  | 0.621 | 0.478 | 1.79 (1.49-2.14) | **1.16×10^-10^** |
| 41 | chr22_39987017_D | 22 | I2D | 0.591 | 0.594 | 0.93 (0.91-0.95) | **4.73×10^-11^** |  | NA | NA | NA | NA |
| 42 | rs7432375 | 3 | AG | 0.421 | 0.449 | 0.93 (0.91-0.95) | **7.26×10^-11^** |  | 0.791 | 0.435 | 4.89 (4.01-5.99) | **3.16×10^-60^** |
| 43 | chr18_52749216_D | 18 | I2D | 0.589 | 0.567 | 1.07 (1.05-1.09) | **8.03×10^-11^** |  | 0.401 | 0.614 | 0.42 (0.35-0.50) | **9.39×10^-22^** |
| 44 | rs111294930 | 5 | AG | 0.788 | 0.782 | 1.09 (1.06-1.12) | **1.06×10^-10^** |  | 0.996 | 0.719 | 98.03 (37.64-363.52) | **6.71×10^-71^** |
| 45 | rs2973155 | 5 | TC | 0.353 | 0.373 | 0.93 (0.91-0.95) | **1.11×10^-10^** |  | 0.596 | 0.359 | 2.64 (2.19-3.17) | **1.50×10^-26^** |
| 46 | rs5937157 | X | TG | 0.738 | 0.759 | 0.94 (0.92-0.96) | **1.98×10^-10^** |  | 0.880 | 0.751 | 2.42 (1.83-3.22) | **8.34×10^-11^** |
| 47 | rs4523957 | 17 | TG | 0.642 | 0.627 | 1.07 (1.05-1.09) | **2.86×10^-10^** |  | 0.679 | 0.615 | 1.32 (1.09-1.59) | 2.97×10^-3^ |
| 48 | rs12704290 | 7 | AG | 0.111 | 0.123 | 0.90 (0.88-0.93) | **3.33×10^-10^** |  | 0.081 | 0.128 | 0.60 (0.44-0.81) | 5.93×10^-4^ |
| 49 | rs12903146 | 15 | AG | 0.544 | 0.520 | 1.07 (1.05-1.09) | **3.38×10^-10^** |  | 0.282 | 0.569 | 0.30 (0.25-0.36) | **9.42×10^-39^** |
| 50 | rs11210892 | 1 | AG | 0.659 | 0.677 | 0.93 (0.91-0.95) | **3.39×10^-10^** |  | 0.686 | 0.638 | 1.24 (1.02-1.49) | 2.47×10^-2^ |
| 51 | rs2905426 | 19 | TG | 0.611 | 0.628 | 0.93 (0.91-0.95) | **3.63×10^-10^** |  | 0.588 | 0.669 | 0.71 (0.59-0.85) | **1.79×10^-4^** |
| 52 | rs140505938 | 1 | TC | 0.151 | 0.164 | 0.91 (0.89-0.94) | **4.49×10^-10^** |  | 0.001 | 0.169 | 0.00 (0.00-0.03) | **1.13×10^-41^** |
| 53 | chr6_84280274_D | 6 | I2D | 0.524 | 0.505 | 1.07 (1.05-1.09) | **8.15×10^-10^** |  | NA | NA | NA | NA |
| 54 | rs4648845 | 1 | TC | 0.533 | 0.527 | 1.07 (1.05-1.10) | **8.70×10^-10^** |  | 0.832 | 0.476 | 5.46 (4.42-6.76) | **2.17×10^-63^** |
| 55 | rs7405404 | 16 | TC | 0.238 | 0.223 | 1.08 (1.05-1.10) | **1.01×10^-9^** |  | 0.117 | 0.260 | 0.38 (0.29-0.48) | **1.99×10^-16^** |
| 56 | rs6466055 | 7 | AC | 0.350 | 0.332 | 1.07 (1.05-1.09) | **1.13×10^-9^** |  | 0.297 | 0.356 | 0.76 (0.63-0.92) | 4.58×10^-3^ |
| 57 | chr1_8424984_D | 1 | I2D | 0.319 | 0.301 | 1.07 (1.05-1.10) | **1.17×10^-9^** |  | 0.082 | 0.320 | 0.19 (0.14-0.25) | **2.05×10^-40^** |
| 58 | rs4766428 | 12 | TC | 0.481 | 0.474 | 1.07 (1.05-1.09) | **1.40×10^-9^** |  | 0.917 | 0.453 | 13.25 (10.22-17.33) | **5.14×10^-111^** |
| 59 | rs10520163 | 4 | TC | 0.493 | 0.470 | 1.07 (1.04-1.09) | **1.47×10^-9^** |  | 0.253 | 0.496 | 0.34 (0.28-0.42) | **1.87×10^-29^** |
| 60 | rs117074560 | 6 | TC | 0.042 | 0.048 | 0.85 (0.81-0.90) | **1.64×10^-9^** |  | 0 | 0.038 | 0.00 (0.00-0.10) | **4.68×10^-10^** |
| 61 | rs6002655 | 22 | TC | 0.456 | 0.443 | 1.07 (1.04-1.09) | **1.71×10^-9^** |  | 0.662 | 0.425 | 2.64 (2.20-3.18) | **1.87×10^-26^** |
| 62 | chr2_146436222_I | 2 | I2D | 0.176 | 0.163 | 1.09 (1.06-1.12) | **1.81×10^-9^** |  | 0.095 | 0.185 | 0.46 (0.35-0.61) | **6.74×10^-9^** |
| 63 | rs9420 | 11 | AG | 0.327 | 0.311 | 1.07 (1.05-1.09) | **2.24×10^-9^** |  | 0.097 | 0.302 | 0.25 (0.19-0.32) | **1.22×10^-30^** |
| 64 | rs11027857 | 11 | AG | 0.515 | 0.499 | 1.06 (1.04-1.09) | **2.55×10^-9^** |  | 0.351 | 0.562 | 0.42 (0.35-0.51) | **2.54×10^-21^** |
| 65 | rs1498232 | 1 | TC | 0.311 | 0.296 | 1.07 (1.05-1.09) | **2.86×10^-9^** |  | 0.295 | 0.307 | 0.94 (0.78-1.15) | 5.40×10^-1^ |
| 66 | rs3735025 | 7 | TC | 0.657 | 0.642 | 1.07 (1.04-1.09) | **3.28×10^-9^** |  | 0.679 | 0.640 | 1.19 (0.98-1.43) | 6.90×10^-2^ |
| 67 | rs11139497 | 9 | AT | 0.346 | 0.337 | 1.07 (1.05-1.09) | **3.61×10^-9^** |  | 0.674 | 0.338 | 4.04 (3.34-4.89) | **2.80×10^-51^** |
| 68 | rs77149735 | 1 | AG | 0.023 | 0.019 | 1.32 (1.20-1.44) | **3.73×10^-9^** |  | 0 | 0.022 | 0.00 (0.00-0.18) | **2.35×10^-6^** |
| 69 | rs56205728 | 15 | AG | 0.291 | 0.274 | 1.07 (1.05-1.10) | **4.18×10^-9^** |  | 0.120 | 0.288 | 0.34 (0.26-0.43) | **7.50×10^-21^** |
| 70 | rs2053079 | 19 | AG | 0.755 | 0.769 | 0.93 (0.91-0.95) | **4.49×10^-9^** |  | 0.820 | 0.741 | 1.60 (1.29-2.00) | **1.49×10^-5^** |
| 71 | rs16867576 | 5 | AG | 0.889 | 0.883 | 1.10 (1.07-1.14) | **4.61×10^-9^** |  | 0.969 | 0.890 | 3.91 (2.57-6.08) | **3.04×10^-12^** |
| 72 | rs4330281 | 3 | TC | 0.479 | 0.480 | 0.94 (0.92-0.96) | **4.64×10^-9^** |  | 0.023 | 0.519 | 0.02 (0.01-0.03) | **1.55×10^-138^** |
| 73 | rs3849046 | 5 | TC | 0.542 | 0.523 | 1.06 (1.04-1.09) | **4.67×10^-9^** |  | 0.215 | 0.509 | 0.26 (0.22-0.32) | **8.60×10^-43^** |
| 74 | rs2693698 | 14 | AG | 0.412 | 0.418 | 0.94 (0.92-0.96) | **4.80×10^-9^** |  | 0.032 | 0.466 | 0.04 (0.03-0.05) | **1.31×10^-112^** |
| 75 | rs2332700 | 14 | CG | 0.262 | 0.249 | 1.07 (1.05-1.10) | **4.86×10^-9^** |  | 0.246 | 0.247 | 1.00 (0.81-1.23) | 9.80×10^-1^ |
| 76 | rs1501357 | 5 | TC | 0.794 | 0.802 | 0.93 (0.90-0.95) | **5.05×10^-9^** |  | 0.538 | 0.816 | 0.26 (0.21-0.32) | **1.08×10^-40^** |
| 77 | rs6984242 | 8 | AG | 0.586 | 0.600 | 0.94 (0.92-0.96) | **5.97×10^-9^** |  | 0.617 | 0.603 | 1.06 (0.88-1.27) | 5.29×10^-1^ |
| 78 | chr1_243881945_I | 1 | I2D | 0.638 | 0.619 | 1.07 (1.05-1.09) | **6.53×10^-9^** |  | 0.425 | 0.656 | 0.39 (0.32-0.47) | **1.99×10^-25^** |
| 79 | rs79212538 | 5 | TG | 0.051 | 0.046 | 1.16 (1.10-1.21) | **7.00×10^-9^** |  | 0 | 0.036 | 0.00 (0.00-0.10) | **1.36×10^-9^** |
| 80 | rs3768644 | 2 | AG | 0.097 | 0.101 | 0.90 (0.87-0.94) | **7.39×10^-9^** |  | 0.034 | 0.116 | 0.27 (0.17-0.40) | **1.98×10^-12^** |
| 81 | rs77502336 | 11 | CG | 0.337 | 0.322 | 1.07 (1.04-1.09) | **7.54×10^-9^** |  | 0.437 | 0.300 | 1.81 (1.50-2.18) | **2.29×10^-10^** |
| 82 | rs6704641 | 2 | AG | 0.819 | 0.805 | 1.08 (1.05-1.11) | **8.33×10^-9^** |  | 0.536 | 0.831 | 0.23 (0.19-0.29) | **4.95×10^-46^** |
| 83 | rs59979824 | 2 | AC | 0.322 | 0.337 | 0.94 (0.92-0.96) | **8.41×10^-9^** |  | 0.412 | 0.336 | 1.38 (1.15-1.66) | 4.45×10^-4^ |
| 84 | rs1106568 | 4 | AG | 0.747 | 0.761 | 0.93 (0.91-0.96) | **9.47×10^-9^** |  | 0.733 | 0.750 | 0.91 (0.74-1.12) | 3.73×10^-1^ |
| 85 | rs10503253 | 8 | AC | 0.223 | 0.219 | 1.07 (1.05-1.10) | **1.06×10^-8^** |  | 0.352 | 0.217 | 1.96 (1.60-2.41) | **1.61×10^-11^** |
| 86 | rs10043984 | 5 | TC | 0.266 | 0.252 | 1.07 (1.05-1.09) | **1.09×10^-8^** |  | 0.141 | 0.261 | 0.46 (0.37-0.58) | **1.49×10^-11^** |
| 87 | rs11685299 | 2 | AC | 0.313 | 0.326 | 0.94 (0.92-0.96) | **1.12×10^-8^** |  | 0.364 | 0.330 | 1.16 (0.96-1.40) | 1.08×10^-1^ |
| 88 | rs7819570 | 8 | TG | 0.187 | 0.174 | 1.08 (1.05-1.11) | **1.22×10^-8^** |  | 0.103 | 0.199 | 0.46 (0.36-0.60) | **2.05×10^-9^** |
| 89 | rs715170 | 18 | TC | 0.261 | 0.275 | 0.94 (0.91-0.96) | **1.27×10^-8^** |  | 0.165 | 0.286 | 0.49 (0.39-0.61) | **6.58×10^-11^** |
| 90 | rs9922678 | 16 | AG | 0.299 | 0.281 | 1.07 (1.04-1.09) | **1.28×10^-8^** |  | 0.123 | 0.321 | 0.30 (0.23-0.38) | **1.08×10^-26^** |
| 91 | rs78322266 | 18 | TG | 0.035 | 0.029 | 1.19 (1.12-1.26) | **1.32×10^-8^** |  | 0.001 | 0.033 | 0.03 (0.00-0.18) | **3.01×10^-8^** |
| 92 | rs2068012 | 14 | TC | 0.760 | 0.771 | 0.93 (0.91-0.96) | **1.41×10^-8^** |  | 0.756 | 0.780 | 0.87 (0.70-1.08) | 1.95×10^-1^ |
| 93 | rs832187 | 3 | TC | 0.607 | 0.615 | 0.94 (0.92-0.96) | **1.43×10^-8^** |  | 0.468 | 0.598 | 0.59 (0.49-0.71) | **4.80×10^-9^** |
| 94 | rs8044995 | 16 | AG | 0.173 | 0.162 | 1.08 (1.05-1.11) | **1.51×10^-8^** |  | 0.099 | 0.161 | 0.57 (0.43-0.75) | **3.72×10^-5^** |
| 95 | chr2_149429178_D | 2 | I2D | 0.955 | 0.961 | 0.86 (0.81-0.90) | **1.59×10^-8^** |  | 1.000 | 0.947 | Inf (14.61-Inf) | **1.52×10^-13^** |
| 96 | rs8082590 | 17 | AG | 0.611 | 0.614 | 0.94 (0.92-0.96) | **1.77×10^-8^** |  | 0.095 | 0.623 | 0.06 (0.05-0.08) | **1.10×10^-134^** |
| 97 | rs12148337 | 15 | TC | 0.478 | 0.465 | 1.06 (1.04-1.08) | **1.79×10^-8^** |  | 0.534 | 0.482 | 1.23 (1.03-1.47) | 2.05×10^-2^ |
| 98 | rs12325245 | 16 | AT | 0.849 | 0.859 | 0.92 (0.89-0.95) | **1.87×10^-8^** |  | 0.851 | 0.847 | 1.03 (0.80-1.33) | 7.89×10^-1^ |
| 99 | rs2239063 | 12 | AC | 0.729 | 0.714 | 1.07 (1.04-1.09) | **1.93×10^-8^** |  | 0.737 | 0.699 | 1.21 (0.99-1.48) | 5.62×10^-2^ |
| 100 | rs12522290 | 5 | CG | 0.840 | 0.830 | 1.08 (1.05-1.12) | **1.99×10^-8^** |  | 0.997 | 0.817 | 74.85 (25.08-366.39) | **5.42×10^-44^** |
| 101 | rs10803138 | 1 | AG | 0.232 | 0.238 | 0.93 (0.91-0.96) | **2.03×10^-8^** |  | 0.028 | 0.267 | 0.08 (0.05-0.12) | **6.12×10^-52^** |
| 102 | rs73229090 | 8 | AC | 0.107 | 0.116 | 0.91 (0.88-0.94) | **2.10×10^-8^** |  | 0 | 0.113 | 0.00 (0.00-0.03) | **3.67×10^-28^** |
| 103 | rs324017 | 12 | AC | 0.293 | 0.309 | 0.94 (0.92-0.96) | **2.13×10^-8^** |  | 0.464 | 0.283 | 2.19 (1.82-2.65) | **4.72×10^-17^** |
| 104 | rs12845396 | X | AT | 0.739 | 0.753 | 0.95 (0.93-0.97) | **2.21×10^-8^** |  | 0.703 | 0.766 | 0.72 (0.57-0.91) | 2.18×10^-2^ |
| 105 | rs55833108 | 10 | TG | 0.208 | 0.196 | 1.08 (1.05-1.10) | **2.23×10^-8^** |  | 0.082 | 0.187 | 0.39 (0.29-0.52) | **6.25×10^-12^** |
| 106 | rs9841616 | 3 | AT | 0.158 | 0.167 | 0.93 (0.90-0.95) | **2.35×10^-8^** |  | 0.133 | 0.179 | 0.70 (0.55-0.90) | 4.45×10^-3^ |
| 107 | rs76869799 | 1 | CG | 0.959 | 0.964 | 0.85 (0.80-0.90) | **2.64×10^-8^** |  | 1.000 | 0.958 | Inf (11.34-Inf) | **5.53×10^-11^** |
| 108 | rs1339227 | 6 | TC | 0.347 | 0.368 | 0.94 (0.92-0.96) | **2.69×10^-8^** |  | 0.401 | 0.342 | 1.29 (1.07-1.55) | 6.28×10^-3^ |
| 109 | chr7_24747494_D | 7 | DI3 | 0.104 | 0.096 | 1.10 (1.06-1.14) | **2.85×10^-8^** |  | 0.056 | 0.101 | 0.52 (0.36-0.74) | **1.31×10^-4^** |
| 110 | rs4388249 | 5 | TC | 0.212 | 0.213 | 1.08 (1.05-1.10) | **3.05×10^-8^** |  | 0.622 | 0.154 | 9.02 (7.26-11.26) | **5.89×10^-103^** |
| 111 | rs215411 | 4 | AT | 0.331 | 0.314 | 1.06 (1.04-1.09) | **3.06×10^-8^** |  | 0.071 | 0.305 | 0.18 (0.13-0.23) | **4.69×10^-41^** |
| 112 | rs11740474 | 5 | AT | 0.601 | 0.621 | 0.94 (0.92-0.96) | **3.15×10^-8^** |  | 0.952 | 0.588 | 13.96 (10.15-19.59) | **5.05×10^-84^** |
| 113 | rs1023500 | 22 | TC | 0.817 | 0.810 | 1.08 (1.05-1.10) | **3.43×10^-8^** |  | 0.877 | 0.799 | 1.79 (1.40-2.30) | **2.15×10^-6^** |
| 114 | rs12421382 | 11 | TC | 0.318 | 0.334 | 0.94 (0.92-0.96) | **3.70×10^-8^** |  | 0.232 | 0.344 | 0.58 (0.47-0.70) | **3.03×10^-8^** |
| 115 | rs211829 | 7 | TC | 0.641 | 0.628 | 1.06 (1.04-1.08) | **3.71×10^-8^** |  | 0.844 | 0.577 | 3.98 (3.21-4.95) | **4.80×10^-40^** |
| 116 | rs679087 | 12 | AC | 0.324 | 0.337 | 0.94 (0.92-0.96) | **3.91×10^-8^** |  | 0.281 | 0.353 | 0.72 (0.59-0.87) | 5.03×10^-4^ |
| 117 | rs75575209 | 2 | AT | 0.904 | 0.913 | 0.90 (0.87-0.94) | **3.95×10^-8^** |  | 0.980 | 0.916 | 4.56 (2.75-7.90) | **6.76×10^-11^** |
| 118 | rs7801375 | 7 | AG | 0.146 | 0.152 | 0.92 (0.90-0.95) | **4.42×10^-8^** |  | 0.040 | 0.153 | 0.23 (0.16-0.33) | **6.44×10^-18^** |
| 119 | rs14403 | 1 | TC | 0.207 | 0.222 | 0.93 (0.91-0.96) | **4.42×10^-8^** |  | 0.257 | 0.207 | 1.33 (1.07-1.64) | 7.62×10^-3^ |
| 120 | rs6670165 | 1 | TC | 0.196 | 0.184 | 1.08 (1.05-1.10) | **4.45×10^-8^** |  | 0.190 | 0.201 | 0.94 (0.75-1.17) | 5.59×10^-1^ |
| 121 | rs7523273 | 1 | AG | 0.695 | 0.685 | 1.06 (1.04-1.09) | **4.47×10^-8^** |  | 0.906 | 0.670 | 4.73 (3.67-6.14) | **2.56×10^-38^** |
| 122 | rs7267348 | 20 | TC | 0.741 | 0.754 | 0.94 (0.92-0.96) | **4.56×10^-8^** |  | 0.762 | 0.733 | 1.17 (0.95-1.44) | 1.30×10^-1^ |
| 123 | rs4240748 | 12 | CG | 0.358 | 0.366 | 0.94 (0.92-0.96) | **4.59×10^-8^** |  | 0.222 | 0.374 | 0.48 (0.39-0.58) | **1.05×10^-13^** |
| 124 | rs2909457 | 2 | AG | 0.568 | 0.593 | 0.94 (0.93-0.96) | **4.62×10^-8^** |  | 0.893 | 0.583 | 5.94 (4.68-7.60) | **3.49×10^-56^** |
| 125 | rs56873913 | 19 | TG | 0.775 | 0.766 | 1.07 (1.05-1.10) | **4.69×10^-8^** |  | 0.807 | 0.782 | 1.16 (0.93-1.45) | 1.78×10^-1^ |
| 126 | rs190065944 | 15 | AG | 0.274 | 0.260 | 1.08 (1.05-1.11) | **4.71×10^-8^** |  | NA | NA | NA | NA |
| 127 | rs10860964 | 12 | TC | 0.650 | 0.646 | 1.06 (1.04-1.08) | **4.84×10^-8^** |  | 0.845 | 0.621 | 3.33 (2.68-4.15) | **6.26×10^-30^** |
| 128 | chr5_140143664_I | 5 | I12D | 0.486 | 0.475 | 1.06 (1.04-1.08) | **4.85×10^-8^** |  | 0.473 | 0.467 | 1.02 (0.86-1.23) | 7.87×10^-1^ |

GWAS: genome-wide association study, Chr: chromosome, EAS: East Asian, EUR: European, I: insertion, D: deletion
